# On the enigmatic scent glands of dyspnoan harvestmen (Arachnida, Opiliones): first evidence for the production of volatile secretions

**DOI:** 10.1007/s00049-014-0146-5

**Published:** 2014-01-31

**Authors:** Günther Raspotnig, Miriam Schaider, Edith Stabentheiner, Hans-Jörg Leis, Ivo Karaman

**Affiliations:** 1 Institute of Zoology, Karl-Franzens-University, Universitätsplatz 2, 8010 Graz, Austria; 2 Research Unit of Osteology and Analytical Mass Spectrometry, Medical University, University Children’s Hospital, Auenbruggerplatz 30, 8036 Graz, Austria; 3 Department of Limnology and Bio-Oceanography, University of Vienna, Althanstraße 14, 1090 Vienna, Austria; 4 Institute of Plant Sciences, Karl-Franzens-University, Schubertstraße 51, 8010 Graz, Austria; 5 Department of Biology and Ecology, Faculty of Science, University of Novi Sad, Trg Dositeja Obradovica 2, 2100 Novi Sad, Serbia

**Keywords:** *Carinostoma*, Nemastomatidae, Dyspnoi, Ethyl ketone, Methyl ketone, Naphthoquinones

## Abstract

While considerable knowledge on the chemistry of the scent gland secretions from the opilionid suborders Laniatores and Cyphophthalmi has been compiled, it is the Palpatores (Eupnoi and Dyspnoi) where chemical data are scarce. In particular, the Dyspnoi have remained nearly unstudied, mainly due to their reported general reluctance to release secretions as well as to the phenomenon of production of insoluble—and inaccessible—solid secretion. We here show that at least certain nemastomatid Dyspnoi, namely all three species of genus *Carinostoma*, indeed produce a volatile secretion, comprising octan-3-one, 6-methyl-5-hepten-2-one and acetophenone in species-specific combinations. In all *Carinostoma* spp., these volatiles are embedded in a semi-volatile, naphthoquinone matrix (mainly 1,4-naphthoquinone and 6-methyl-1,4-naphthoquinone). In detail, acetophenone and traces of naphthoquinones characterize the secretions of *Carinostoma carinatum*. A mixture of octan-3-one, 6-methyl-5-hepten-2-one and large amounts of naphthoquinones were found in *C. elegans*, and 6-methyl-5-hepten-2-one together with small amounts of naphthoquinones in the secretions of *C. ornatum*. So far, exclusively naphthoquinones had been reported from a single dyspnoan hitherto studied, *Paranemastoma quadripunctatum*.

## Introduction

Prosomal scent glands (syn. defense glands, stink glands) characterize all species of harvestmen, constituting one of their most important complex synapomorphic characters (Martens 1978; Gnaspini and Hara 2007). Scent glands show major modifications across opilionid taxa and may be developed as (1) pronounced organs for chemical defense as in the Laniatores and in the Cyphophthalmi (e.g., Duffield et al. 1981; Eisner et al. 1971, 1977, 2004; Gutjahr et al. 2006; Raspotnig et al. 2005) or (2) as rather inconspicuous glands as in many Palpatores (e.g., Schaider and Raspotnig 2009; Schaider et al. 2010). From the first group, a large number of scent gland-derived products have been identified, including naphthoquinones and methyl ketones from Cyphophthalmi, alkylated benzoquinones and vinyl ketones from gonyleptoid Laniatores, phenolics from some lower grassatorean Laniatores, and small tobacco alkaloids from travunioid Insidiatores (for a summary see Raspotnig 2012). It is, however, the Palpatores (Eupnoi and Dyspnoi) where scent glands are poorly investigated, but most heterogeneously developed, displaying transitions from the possibly plesiomorphic type of typical defense glands as still present in some Eupnoi (Holmberg 1970; Meinwald et al. 1971; Jones et al. 1976, 1977; Wiemer et al. 1978) toward more cryptic and apparently dysfunctional organs as in most Dyspnoi (see Schaider and Raspotnig 2009). First chemical data reflect this heterogeneity as well: sclerosomatid Eupnoi, at least some leiobunines, show highly volatile, acyclic ethyl ketone-rich exudates (Ekpa et al. 1985), whereas the secretions of phalangiid Eupnoi may rely on naphthoquinones (Wiemer et al. 1978). Meanwhile there is evidence for further, enormous, still undescribed chemical diversity in eupnoan subgroups (Raspotnig 2012).

On the other hand, the scent glands of dyspnoans have remained even more enigmatic: In many species, such as in all Trogulidae but also in many Ischyropsalidoidea, no secretion release can be detected at all (e.g., Pabst 1953). In trogulids, ozopores may even be hidden and covered by cuticular folds and other structures, making rapid emission of secretions impossible (Schaider and Raspotnig 2009). Paralleling these observations, a unique trend toward solidification of secretions can be observed: The secretion products of *Ischyropsalis* and *Trogulus*, for instance, were described as solids—as crystals (Juberthie et al. 1991) or as solid balls of unknown chemistry (Schaider and Raspotnig 2009). It is still unknown how such products—if at all—are eventually released to the body outside, even though a kind of sublimation, followed by “exhalation from the ozopore” has speculatively been proposed (Juberthie et al. 1991; Gnaspini and Hara 2007). In such species, the defensive role of the glands may have been lost and replaced by other functions such as territorial marking, intraspecific communication or pronounced chemical protection against microorganisms (Holmberg 1986; Juberthie et al. 1991).

However, at least certain Dyspnoi are able to release liquid secretions as well: as a first example for dyspnoan chemistry, the scent gland secretion of the nemastomatid *Paranemastoma quadripunctatum* was reported to contain semi-volatiles such as 1,4-naphthoquinone and 6-methyl-1,4-naphthoquinone along with a series of basically non-volatile anthraquinones (Raspotnig et al. 2010). All these compounds again form solids at room temperature and ambient pressure conditions and may be stored in their solid state in the scent gland reservoirs, but are liquefied by mixing up with enteric fluid in the course of the emission process (Schaider et al. 2011).

We here report on a further chemical class of components in dyspnoid secretions, indeed volatile compounds that characterize the scent secretions of all species of genus *Carinostoma*.

## Materials and methods

### Collection of specimens

**Fig. 1:**
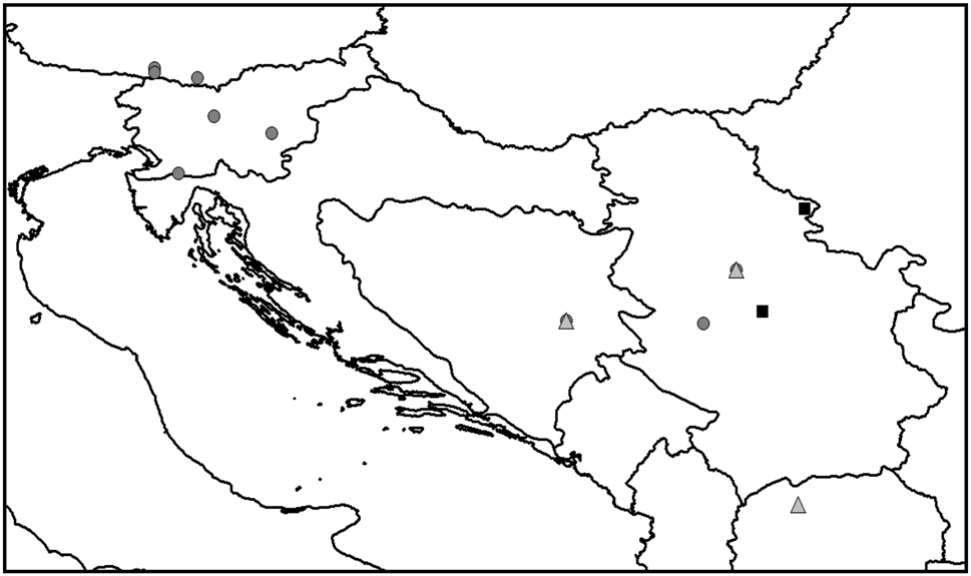
Collection sites for populations of *C. ornatum* (*gray triangles*), *C. elegans* (*black squares*), and *C. carinatum* (*gray circles*). Note the sympatric occurrence of *C. ornatum* and *C. carinatum* at two collection sites (one location in Bosnia-Herzegovina, another one in Serbia). For details see Table 1

**Fig. 2:**
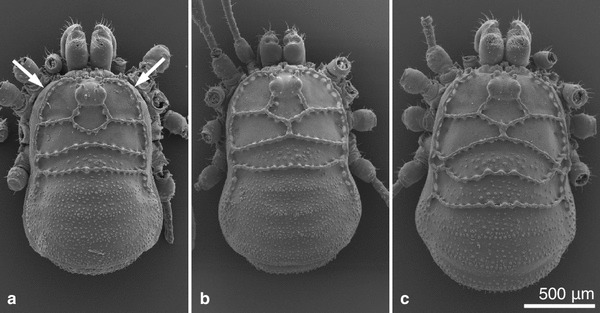
The study objects: *Dorsal view* (SEM) of each male individual of **a** *C. ornatum*, **b** *C. elegans*, and **c** *C. carinatum*. Note the differences in the dorsal “carinae” (rows of anvil-shaped bridge thorns: compare Karaman 1995). *Arrows point* to the site of ozopores

All specimens were collected by hand or by sieving leaf litter prior to extraction of specimens in a Berlese-Tullgren apparatus. All specimens were collected alive. *Carinostoma carinatum*, in all 65 adult individuals of both sexes, was collected from several populations in Austria, Slovenia, Bosnia-Herzegovina, and Serbia. *C. elegans* (28 individuals) was from two populations in Serbia, and *C. ornatum* (44 individuals) from different populations in Bosnia-Herzegovina, Serbia, and Macedonia (Fig. 1; Table 1). From the location in Bosnia-Herzegovina and from one location in Serbia, the sympatric occurrence of *C. ornatum* with *C. carinatum* was recorded (Fig. 1). According to the current view on *Carinostoma* (e.g., Martens 1978), we thus collected all valid species of this genus. We, however, did not include representatives of “*C. elegans batorligetiense”* which was handled as a subspecies by Loksa (1991) but considered to be a color variation of *C. elegans* by others (e.g., Szalay 1952; Martens 1978). *Carinostoma carinatum* shows a large distributional area from the eastern Alps to the western Balkans; *C. ornatum* follows in the south, partly overlapping with south-eastern populations of *C. carinatum*, and extends toward the south-eastern Balkans. *C. elegans* is found from the Carpatho-Ukraine and Southern Slovakia across eastern Hungary and Romania to the north-eastern parts of Serbia and to Bulgaria. Species determination of collected individuals was carried out according to Martens (1978) and Karaman (1995), mainly on the basis of the characteristic rows of bridgethorns on the dorsum of the cephalothorax (Fig. 2).

**Table 1:** Collection of *Carinostoma* species and corresponding extracts

Species	Populations, location (coordinates, altitude)	Date of collection	Extracts^a^
*C. carinatum*			
Austria	1. Carinthia, Villach, Eichholzgraben (N 46°38′, E 13°50′, 590 m, C. Komposch leg.)	5 April 2010	Pool (4 ind.), pool (5ind.)
	2. Carinthia, Villach, Graschelitzen (N 46°34′35′′, E 13°49′57′′, 600 m, A. Platz leg.)	8 October 2012	1♂, 1♀
	3. Carinthia, near Ferlach, Rauth, Dixer (N 46°31′11.46″, E 14°19′56.89″, 583 m, G. Raspotnig leg.)	30 April 2010	Pool (4 ind.), pool (4 ind.)
	4. Carinthia, near Ferlach, Rauth, Moatsche (N 46°31′24.11′’, E 14°19′42.69″, 509 m, G. Raspotnig leg.)	6 August 201	1♀
Slovenia	5. Near Ljubljana (N 46°6′19.10′′, E 14°31′5.60′′, 323 m, S. Huber leg.)	5 September 2012	6♂, 7♀
	6. Laknice (N 45°56′03′′, E 15°11′39′′, 237 m, T. Novak leg.)	26 October 2013	4♂, 5♀
	7. Poljane pri Podgradu (N 45°29′′57′′, E 14°06′′44′′, 600–700 m, S. Novak & T. Novak leg.)	28 October 2013	2♂, 1♀
Bosnia-Herzegovina	8. Republika Srpska, Romanija Mt, Pale, Kadino selo (N 43°55′29′′, E 18°35′43′′, 1,000 m, I. Karaman leg.)	14 August 2011	4♂, 1♀
Serbia	9. Western Serbia, Čačak, Ovčar banja (N 43°53′18.37′′, E 20°11′19.31′′, 370 m, G. Raspotnig, P. Föttinger and I. Karaman leg.)	20 May 2009	1(?)
	10. Kosmaj, near monument (44°28′7.28″N 20°34′20.29″E, 550 m, I. Karaman and S. Ivković leg.)	26 October 2013	10♂, 4♀
*C. elegans*			
Serbia	11. Vršac, Vršački breg (45° 7′14.26″N, 21°22′18.42″E, 190 m, I. Karaman & S. Ivković leg.)	2 November 2013	1♂
	12. Kragujevac, Šumarice (44° 1′4.72″N, 20°52′45.06″E, 240 m, I. Karaman & S. Ivković leg.)	5 November 2013	17♂, 10♀
*C. ornatum*			
Bosnia-Herzegovina	13. Republika Srpska, Romanija Mt, Pale, Kadino selo (N 43°55′29′’, E 18°35′43′’, 1 000 m, I. Karaman leg.)	14 August 2011	3♂, 2♀
Serbia	14. Kosmaj, Monastir Tresije (N 44°28′25.35″, E 20°34′6.42″, 400 m, I. Karaman & M. Horvatović leg.)	30 September 2012	6♂, 3♀
	15. Kosmaj, near monument (N 44°28′7.28″ E 20°34′20.29″ 550 m, I. Karaman S. Ivković leg.)	26 October 2013	8♂, 4♀
Macedonia	16. Skopje, Matka, near old church St. Nedela (N 41°56′56.94′′, E 21°17′19.2′′, 726 m, M. Komnenov leg.)	6 October 2013	7♂, 11♀

^a^Extracts are individual extracts except for four pools, as indicated

### Extraction and analysis of secretions

Scent gland secretion was obtained by whole body extraction of living individuals in 50 μl of hexane or methylene chloride for 30 min as already described and standardized for other opilionids (e.g., Raspotnig et al. 2010). In total, 124 extracts were prepared, mostly extracts of single individuals (adults), and in some cases also pooled extracts, as summarized in Table 1. For extracts of single specimens, the gender was determined as well (Table 1). Aliquots of extracts (1.5 μl) were subjected to gas chromatographic–mass spectrometric analysis, using a trace gas chromatograph coupled to a DSQ I mass spectrometer (MS), both from Thermo (Vienna, Austria). The GC was equipped with a ZB-5 fused silica capillary column (30 m × 0.25 mm i.d., 0.25 μm film thickness, Phenomenex, Germany). Injection was splitless with helium (at 1.2 ml min^−1^) as a carrier gas. The temperature of the GC oven was raised from 50 °C (1 min) to 300 °C at 10 °C min^−1^, and then held for 5 min at 300 °C. The ion source of the MS and the transfer line were kept at 200 and 310 °C, respectively. Electron impact (EI) spectra were recorded at 70 eV. Gas chromatographic retention indices (RI) of extract components were calculated using an alkane standard mixture, following the formula RI_x_ = 100*n*_0_ + (100*t*_x_ − 100*tn*_0_)/(*tn*_1_ − *tn*_0_), with x: target compound; *t*_x_: retention time of target compound; *n*_0_: number of carbon atoms in the alkane directly eluting before x; *tn*_0_: retention time of alkane directly eluting before x; *tn*_1_: retention time of alkane directly eluting after x.

### Reference compounds and derivatization

Authentic standards of *n*-alkanes (C_7_–C_36_), acetophenone, octan-3-one, 5-methyl-heptan-3-one, 6-methyl-5-hepten-2-one, 1,4-naphthoquinone and 2-methoxy-1,4-naphthoquinone for a comparison of gas chromatographic–mass spectrometric data to components found in the *Carinostoma*-secretion were purchased from Sigma (Vienna, Austria). 6-Methyl-1,4-naphthoquinone was prepared according to Bruce and Thomson (1952); as oxidizing reagent we used CAN (=cerium IV ammonium nitrate from Sigma, Austria). For two further compounds, namely 2-methoxy-6-methyl-1,4-naphthoquinone and 4-chloro-1,2-naphthoquinone, we used known natural sources for reference, namely the secretions of *P. quadripunctatum* (Raspotnig et al. 2010) and *Cyphophthalmus* (formerly *Siro*) *duricorius* (Raspotnig et al. 2005). *O*-Methyl oximes of ketones in the *Carinostoma*-extracts were prepared by adding 200 μl of methoxamine (MOX) reagent (=2 % methoxyamine-hydrogen chloride in pyridine, Thermo Scientific, Vienna, Austria) to 60 μl of ketone-rich extracts in hexane, followed by incubation at 70° for 1 h. The products were washed twice with water, dissolved in 60 μl of hexane, and an aliquot (1.5 μl) was directly used for GC–MS.

### Scanning electron microscopy and histology

For scanning electron microscopy (SEM), specimens were fixed in Bouin, washed, dehydrated, air dried, and mounted on aluminum stubs prior to sputter coating with gold (AGAR sputtercoater, Gröpl, Tulln, Austria). Micrographs (SEM) were taken with a Philips XL30 ESEM (Philips/FEI, Vienna, Austria) at high vacuum mode and 20 kV accelerating voltage. For semithin cross sections (2.5 μm), specimens were fixed in Bouin, washed, dehydrated, and embedded in LR-White resin (soft grade; Gröpl, Tulln, Austria). Sections were prepared on a Leica Supercut rotary microtome (Vienna, Austria) and stained with toluidine blue O.

### Evaluation of secretion profiles and multivariate statistics

Secretion profiles were evaluated by integration of peak areas of each component of the secretion and by calculation of the relative abundance of these peaks (expressed in % peak area of whole secretion). A statistical comparison of individual secretion profiles of all three species of *Carinostoma* was performed in PAST (PAlaeontological STatistics, version 2.17) and was based on the method of 2D non-metric multidimensional scaling (2D-nMDS) using the Gower coefficient of dissimilarity (Gower 1967).

## Results

### Scent gland morphology

**Fig. 3:**
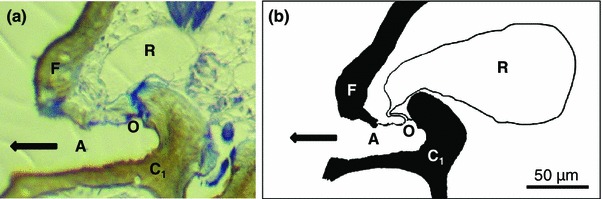
Morphology of scent glands in *Carinostoma*. **a** Cross section through an individual of *C. carinatum*, at the height of coxae I, showing left scent gland reservoir (*R*), ventrally directed ozopore (*O*), and covering lateral fold of the cephalothorax (*F*). Note that an atrium-like cavity (*A*) is formed at the lateral fold and the coxa I; this atrium is laterally open (*arrow*). **b** Schematic drawing of the same structure

The paired scent glands in all species of *Carinostoma* are represented by small glandular sacs in the prosoma, basically comprising intima-lined secretion reservoirs (left reservoir *R* of an individual is shown in Fig. 3) opening into ozopores. Ozopores (=scent gland orifices *O*, Fig. 3; one at each side of the prosoma) are located near dorsal to coxae I (white arrows in Fig. 2). Ozopores are not visible from the outside, but hidden under a laterally protruding fold of the prosomal integument (*F* in Fig. 3). They are directly situated within the underside of these folds, hence being directed ventrally (Fig. 3). All products released from the scent glands must be emitted into the cavity roved over by these folds first (“atrium” *A* in Fig. 3). The cavities (*A*) themselves are laterally open toward the body outside (arrow in Fig. 3).

### Secretions of *C. ornatum*

**Fig. 4:**
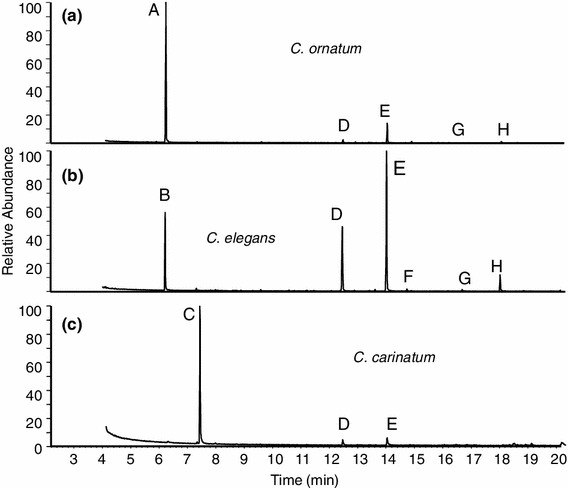
Typical total ion chromatograms from the three species of *Carinostoma*. *Peak A* [6-methyl-5-hepten-2-one], *peak B* [6-methyl-5-hepten-2-one + octan-3-one], peak *C* [acetophenone], *peak D* [1,4-naphthoquinone], *peak E* [6-methyl-1,4-naphthoquinone], *peak F* [4-chloro-1,2-naphthoquinone], *peak G* [2-methoxy-1,4-naphthoquinone], *peak H* [2-methoxy-6-methyl-1,4-naphthoquinone]

From the 44 individual extracts of *C. ornatum* (24♂, 20♀), 22 exhibited large amounts of scent gland secretions, 20 individual extracts showed only traces of scent gland secretions, and three were “empty” (i.e., not showing any peaks). The 22 secretion-containing extracts of individuals irrespective of population allocation consistently exhibited a major peak A, followed by smaller peaks D, E, G, and H (Fig. 4a). Mass spectral data and retention indices of compounds are summarized in Table 2.

**Table 2:** Analytical data to extract components from *C. ornatum*, *C. elegans*, and *C. carinatum*

Peak no.	RI^b^	Mass spectrometric fragmentation (*m*/*z*)	Identified as
A	986	126(10), 111(27), 108(78), 93(26), 83(16), 77(4), 71(26), 69(54), 68(24), 67(29), 58(27), 55(57), 53(11), 43(100), 41(54)	6-Methyl-5-hepten-2-one
B	985	128(10), 126(10), 111(37), 108(96), 99(100), 93(36), 86(8), 85(17), 83(20), 77(5), 73(5), 72(69), 71(79), 69(43), 68(18), 67(21), 58(17), 5 757), 55(34), 43(89), 41(38)	Mixture: octan-3-one + 6-methyl-5-hepten-2-one
C	1068	120(32), 105(100), 77(98), 51(25), 43(10)	Acetophenone
D	1421	159(11), 158(100), 130(33), 104(43), 102(52), 76(39), 75(12), 74(12), 50(15)	1,4-Naphthoquinone
E	1547	173(13), 172(100), 157(9), 144(31), 118(35), 116(39), 115(39), 90(20), 89(26), 63(12)	6-Methyl-1,4-naphthoquinone
F	1606	194(48), 192(100), 166(5), 164(19), 157(50), 138(3), 136(10), 129(60), 104(19), 101(33), 77(5), 76(22), 75(17), 74(11), 50(9)	4-Chloro-1,2-naphthoquinone
G	1782	189(10), 188(100), 173(46), 160(40), 159(38), 158(55), 131(10), 130(18), 104(11), 102(52), 101(13), 89(69), 76(20)	2-Methoxy-1,4-naphthoquinone
H	1912	203(11), 202 (100), 187(48), 174(34), 173(54), 172(25), 145(18), 144(25), 131(20), 116(88), 115(61), 103(90), 89(48), 86(13), 77(46), 69(25), 63(36), 57(38), 51(12)	2-Methoxy-6-methyl-1,4-naphthoquinone

^b^Retention index on a ZB-5 column

Peak A showed the fragmentation pattern of an unsaturated terpenoid with a molecular mass of *M* = 126 (M^+^ at *m*/*z* 126). A fragment at *m*/*z* 58(27) indicated a McLafferty rearrangement product [C_3_H_6_O]^+^ characteristic of a methyl ketone, and the compound was suspected to be sulcatone (=6-methyl-5-hepten-2-one). A detailed comparison of the mass spectral data to the EI spectrum of 6-methyl-5-hepten-2-one from the NIST-library revealed full correspondence, and also the measured retention index matched reported values from literature (measured RI: 986; reported RI: e.g., 987 in Benzo et al. 2007). The identity of the component as 6-methyl-5-hepten-2-one was confirmed by a comparison of gas chromatographic—mass spectrometric data to an authentic sample.

The mass spectra of the two smaller peaks D and E indicated two naphthoquinones with molecular masses of *M* = 158 and *M* = 172, strongly suggesting the presence of 1,4-naphthoquinone (NQ) and 6-methyl-1,4-naphthoquinone (MNQ). The identity of the compounds as NQ and MNQ, respectively, was underlined by RIs completely matching data from literature (RIs at 1421 and 1537 in Raspotnig et al. 2012) and confirmed by comparison to the authentic compounds. Peaks G (M^+^ at *m*/*z* 188) and H (M^+^ at *m*/*z* 202) were proposed to be 2-methoxy-1,4-naphthoquinone and 2-methoxy-6-methyl-1,4-naphthoquinone, respectively. Both compounds were already familiar from a previous study (Raspotnig et al. 2010). 2-Methoxy-1,4-naphthoquinone was finally identified by a comparison to the authentic compound, whereas the identification of 2-methoxy-6-methyl-1,4-naphthoquinone was tentative, based on a comparison to an already known natural source, namely a naphthoquinone-rich extract of *P. quadripunctatum* (Raspotnig et al. 2010).

### Secretions of *C. elegans*

From the 28 individual extracts of *C. elegans* (18♂, 10♀), 17 individuals (12♂, 5♀) contained scent gland secretion, as outlined below. Secretion-loaded extracts consistently showed six peaks (Fig. 4b), five of which matched the peaks of the *ornatum* extracts with respect to their retention time (peaks A, D, E, G, and H in Fig. 4a). An additional, but generally very small peak F was detected, showing an unusual mass spectrum with an M + 2 ion (at *m*/*z* 194) along with a molecular ion at *m*/*z* 192, suggesting a chlorinated compound. The mass spectrum of the compound and its retention index fully matched data reported for 4-chloro-1,2-naphthoquinone (Raspotnig et al. 2005, 2012). For a direct gas chromatographic–mass spectrometric comparison to the authentic compound, we again used a natural source, namely a scent gland-loaded extract from *Cyphophthalmus* *duricorius* for which the compound has already unambiguously been identified (Raspotnig et al. 2005).

**Fig. 5:**
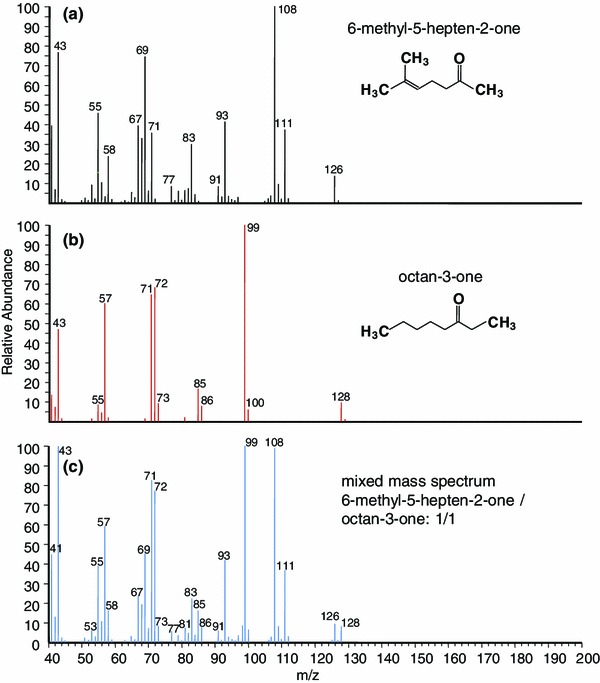
EI-Mass spectra of synthetic **a** 6-methyl-5-hepten-2-one, **b** octan-3-one, and **c** mixed mass spectrum of both (ratio 1:1). Compare to the mass spectrum of *peak B* in Table 2

**Fig. 6:**
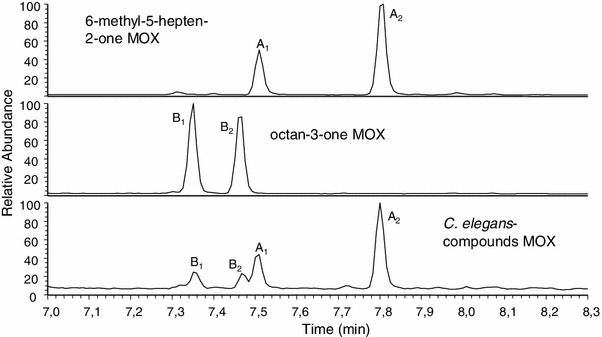
Separation and identification of 6-methyl-5-hepten-2-one/octan-3-one by *O*-methyloximation. **a** MOX treatment of synthetic 6-methyl-5-hepten-2-one leads to two isomeric *O*-methyloximes (*A*_*1*_, *A*_*2*_) in a ratio of 1:2. **b** MOX of synthetic octan-3-one leads to two isomeric *O*-methyloximes (*B*_*1*_, *B*_*2*_) in a ratio of 1:1. **c** MOX of a *C. elegans* extract showing the *O*-methyloxime isomers of both compounds (*A*_*1*_, *A*_*2*_, *B*_*1*_, *B*_*2*_). Mass spectral data to all *O*-methyloximes are summarized in Table 3

Surprisingly, a peak eluting at exactly the same retention time as peak A of the *ornatum* extracts (=designated as peak B in the *elegans* extract) exhibited a mass spectrum different from already identified 6-methyl-5-hepten-2-one. This mass spectrum showed an ion of highest mass at *m*/*z* 128, preceded by an ion at *m*/*z* 126. Since a loss of two hydrogen atoms is highly unusual and an isotopic pattern of a chlorinated compound was not given, this particular pattern indicated either (1) that the ion at *m*/*z* 128 was not the molecular ion but a fragment and that the molecular ion was not detectable under the given EI conditions, or (2) a mixed mass spectrum of two compounds eluting at the same retention time. A mass spectrum of peak B by positive chemical ionization (CI+) showed ions at *m*/*z* 157, 155, 129, and 127, interpreted as M+1 and M+29 adducts (MH^+^ and M–C_2_H_5_^+^). These ions indeed indicated the presence of two different compounds within peak B, thus having molecular masses of *M* = 126 and *M* = 128, respectively. The compounds remained chromatographically inseparable even when changing the chromatographic conditions (lowering of injector temperature, extending the temperature program, etc., had no effect). The mixed EI mass spectrum of peak B, however, exhibited a striking similarity to the spectrum of already known 6-methyl-5-hepten-2-one of the *ornatum* extracts, showing all its fragment ions along with additional fragments at *m*/*z* 57, 72, 85, 99 (base ion), and 128 (peak B, Table 2). This situation tentatively indicated that 6-methyl-5-hepten-2-one was one of the two compounds, hence representing the compound with molecular mass *M* = 126, and that the additional fragments belonged to a second component. These fragments in turn appeared to be consistent with the structure of a saturated C_8_-ethyl ketone, as implied by the intense ion at *m*/*z* 72 (McLafferty rearrangement) and the molecular ion at *m*/*z* 128. Thus, likely candidate structures were essentially limited to octan-3-one and some of its branched isomers, with possible branching sites at C4, C5, and C6 only (C3 bears the keto group; branching at C2 is not supported by the McLafferty fragment at *m*/*z* 72). Moreover, a methyl branching at C4 would lead to 4-methyl-heptan-3-one which shows a clearly different mass spectrum (intense McLafferty product at *m*/*z* 86 and not at *m*/*z* 72) along with a much shorter retention time, as known from a paralleling study on this compound (Raspotnig, unpublished)). Thus, if branched, the branching had to occur in position C5 (=5-methyl-heptan-3-one) or C6 (=6-methyl-heptan-3-one). A literature survey for the RIs of the three candidate compounds revealed that only the RI of octan-3-one (reported RIs on comparable columns are about 986, e.g., RI 985 in Sari et al. 2006; RI 986 in Mohagheghzadeh et al. 2000; RI 987 in Figuérédo et al. 2006) matched the RI of 6-methyl-5-hepten-2-one (measured RI for peak B: 985, Table 2). By contrast, reported RIs of the methyl-branched octanones are distinctly lower (5-methyl-heptan-3-one: RI 944 in, e.g., Morteza-Semnani et al. 2006; 6-methyl-heptan-3-one: RI 941 in, e.g., Usai et al. 2003). Eventually, an authentic sample of octan-3-one led to a peak at the same retention time as 6-methyl-5-hepten-2-one under our given chromatographic conditions (measured RI for authentic octan-3-one: 985). Furthermore, a 1:1 mixture of the two synthetic components (=6-methyl-5-hepten-2-one + octan-3-one) resulted in one single, sharp peak with RI 985, exhibiting a mixed mass spectrum fully consistent with the spectrum of peak B from the *C.* *elegans* extracts (Fig. 5) and thus confirming the identity of octan-3-one as the second compound within peak B. To eventually separate the two compounds in the *C. elegans* extracts and determine their ratio, we prepared their *O*-methyl oximes: derivatization of synthetic octan-3-one with MOX resulted in the generation of two isomeric *O*-methyl oximes (in 1:1 ratio) at RIs 1060 and 1068, respectively, whereas the MOX derivatives of 6-methyl-5-hepten-2-one (arising in 1:2 ratio) were separable at RIs 1071 and 1090 (Table 3; Fig. 6). MOX derivatization of the C. *elegans* extracts revealed exactly the same derivatives, matching in both retention times and mass spectra (Fig. 6). As shown in the synthetic samples, the derivatization to *O*-methyl oximes was quantitative, and the ratio of the derivatives well corresponded to the originally present ratio of the two components. Alternatively, the ratio of the two original components could also be calculated directly from their mixed mass spectrum by a comparison of fragment ions at *m*/*z* 111 (base ion in the spectrum of 6-methyl-5-hepten-2-one under the given conditions) and *m*/*z* 99 (base ion in the spectrum of octan-3-one). For instance, in spectra where these ions showed the same intensity, the ratio of the two components was 1:1. Different ratios of ions *m*/*z* 111 : *m*/*z* 99 directly reflected the quantitative composition of peak B in the *C. elegans* extracts, as subsequently verified by a quantification of the MOX derivatives for all 17 secretion-loaded *C. elegans* extracts. As a result, 6-methyl-5-hepten-2-one was always found to be the major component in the two-compound mixture, on average constituting 2/3 (calculated 67.0 ± 10.0 %) of peak B (max. 80 %, min. 50 %).

**Table 3:** Separation of octan-3-one and 6-methyl-5-hepten-2-one by *O*-methyl oximation

Peak	compound	RI^a^	Mass spectrometric fragmentation (*m*/*z*)
	6-Methyl-5-hepten-2-one (synthetic)	986	126(13), 111(39), 108(100), 93(42), 83(24), 77(6), 71(27), 69(56), 68(23), 67(27), 58(18), 55(38), 53(7), 43(56), 41(32)
A_1_	6-methyl-5-hepten-2-one *O*-methyl oxime isomer 1	1071	155(0.2), 108(3), 97(8), 87(2), 82(100), 79(6), 69(33), 67(32), 55(6), 42(7), 41(17)
A_2_	6-methyl-5-hepten-2-one *O*-methyl oxime isomer 2	1090	155(9), 140(9), 124(16), 112(8), 109(21), 108(17), 107(16), 96(7), 94(9), 87(65), 83(72), 82(100), 69(91), 67(30), 55(32), 42(48), 41(55)
	Octan-3-one (synthetic)	985	128(9), 99(100), 86(8), 85(18), 73(12), 72(78), 71(79), 57(61), 55(9), 43(44)
B_1_	Octan-3-one *O*-methyl oxime isomer 1	1060	157(11), 128(27), 114(25), 110(6), 101(100), 86(6), 82(7), 81(6), 71(16), 70(19), 69(11), 56(20), 42(24)
B_2_	Octan-3-one *O*-methyl oxime isomer 2	1068	157(9), 128(26), 114(23), 110(7), 101(100), 86(5), 82(6), 81(6), 71(16), 70(19), 69(9), 56(22), 42(24)

^a^Retention index on a ZB-5 column

### Secretions of *C. carinatum*

From the 65 individuals of *C. carinatum*, 48 individual extracts and 4 pooled extracts were prepared. All pooled extracts and 14 individual extracts contained scent gland secretion, as outlined below. These extracts basically exhibited three peaks (C, D, and E, Fig. 4c), with the latter two peaks representing the already known naphthoquinones, NQ and MNQ, respectively. Both quinones generally occurred in very small amounts (combined amounts were on average about 11 % of the secretion, see Table 4) and in most individuals even in traces only. In several samples, e.g., in all extracts from the (five) individuals from the population in Bosnia (see Table 1), no trace of them was detected. Peak C, however, was consistently present as the major component of the secretion (Table 4). Its mass spectrum indicated a low molecular weight (M^+^ at *m*/*z* 120) aromatic (phenyl cation at *m*/*z* 77) and completely matched the EI spectrum of acetophenone. The measured retention index was RI 1068, being basically consistent with the RI for acetophenone from literature as well (e.g., RI 1068 in Costa et al. 2009). We finally compared the compound to synthetic acetophenone, showing full correspondence in both retention time and mass spectrum.

**Table 4:** Scent gland secretion profiles of *C. carinatum*, *C. ornatum*, and *C. elegans*

Compounds	*C. carinatum* ^a^	*C. ornatum* ^a^	*C. elegans* ^a^
Octan-3-one	–	–	6.6 ± 2.5
6-Methyl-5-hepten-2-one	–	84.0 ± 19.8	14.9 ± 7.0
Acetophenone	88.8 ± 11.4	–	–
1,4-Naphthoquinone	5.2 ± 5.9	6.6 ± 9.6	24.9 ± 6.2
6-Methyl-1,4-naphthoquinone	5.9 ± 7.3	8.6 ± 9.9	47.1 ± 4.4
4-Chloro-1,2-naphthoquinone	–	Trace	1.1 ± 0.7
2-Methoxy-1,4-naphthoquinone	–	0.2 ± 0.4	0.3 ± 0.3
2-Methoxy-6-methyl-1,4-naphthoquinone	0.1 ± 0.4	0.6 ± 1.0	5.1 ± 1.8

^a^Evaluation of profiles was based on extracts containing amounts of secretions large enough for quantification (=calculation of peak areas), in detail on 14 individual extracts of *C. carinatum*, 22 extracts of *C. ornatum*, and 17 extracts of *C. elegans*. Profiles are given in  % peak area of whole secretion

### Species specificity, sex specificity, interpopulation differences and effect of sympatry

**Fig. 7:**
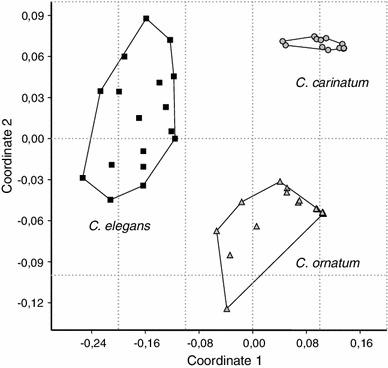
Statistical discrimination of secretion profiles from individuals of different species of *Carinostoma* by multidimensional scaling (2D-nMDS) of Gower dissimilarity. Three homogenous, clearly distinguishable chemical clusters are indicated well corresponding to the three different species

The chemical homogeneity or heterogeneity of the secretions of the three *Carinostoma* species was evaluated by calculating individual secretion profiles based on the relative abundance of single secretion components in the chromatograms. Average profiles for each species are given in Table 4. All profiles (=53 individual extracts; pooled extracts were excluded) were subject to a multivariate statistical comparison by 2D-nMDS, resulting in three distinct chemical clusters corresponding to the three species (Fig. 7). Intraspecifically, (1) no discrimination between the profiles of individuals from different populations was noticed (overlap in nMDS, not shown), and (2) no sexually dimorphic secretions occurred (overlap in nMDS, not shown), all of which underlined the stability of secretion profiles as species-specific characters. In two of our collections (at one locality in Bosnia-Herzegovina and one in Serbia, see Table 1) we noted the sympatric occurrence of *C. ornatum* with *C. carinatum*. Extracts of individuals from these populations either showed 6-methyl-5-hepten-2-one (in *C. ornatum*) or acetophenone (in *C. carinatum*), exactly as described for these species in general. Extracts of *C. ornatum* from sympatric populations seemed to exhibit a slightly lower amount of naphthoquinones (measured as total of NQ and MNQ in the secretion,) than *C. ornatum*-individuals from allopatric populations (sympatric populations—total average content of NQ + MNQ: 10.51 ± 10.86 % of secretion, *n* = 8; allopatric populations—total average content of NQ + MNQ: 19.38 ± 22.01 %, *n* = 13; normal distribution of values not given). A difference between the medians was statistically not significant, as revealed by a two-tailed (Wilcoxon) Mann–Whitney *U* test (*U* = 44, exact *p* = 0.5787). A sympatric occurrence of *C. elegans* and *C. ornatum* was not given in any collection.

### Absolute amounts of secretions

To evaluate how much secretion can be produced and stored, the absolute amounts of MHO in two selected individuals of *C. ornatum* from the Macedonian population were exemplarily quantified, using a calibration curve for authentic MHO. Extracts of the selected specimens (both ♀) had already revealed the largest amounts of secretion of all individuals analyzed (on the basis of peak areas), thus possibly giving an impression of the (near) maximal amount of secretion per individual. We calculated 1.9 μg and 3.0 μg of MHO per individual, respectively. Based on the secretion profiles given above, these amounts would account for about 85 % of the whole secretion (Table 4), thus roughly implying secretion amounts per individual in the lower μg range.

## Discussion

### A novel dyspnoid scent gland “ecotype”

Following a study on *P. quadripunctatum* (Raspotnig et al. 2010), this is the second investigation into the scent gland chemistry of dyspnoan harvestmen, providing first evidence for the production and emission of volatile components—i.e., “scent” in the literal sense. So far, dyspnoans have frequently been regarded as emitting no secretions at all, though scent glands are well developed across all dyspnoan taxa. The reported reluctance or even inability to release secretions may thus refer to certain dyspnoan subgroups only (e.g., Pabst 1953; Shear 2010; see below). In consideration of the results for *Carinostoma* and on the basis of currently available information from literature, at least three ecological types of dyspnoan scent glands can now be distinguished (compare Schaider et al. 2010). (1) A glandular type that relies on the production of solid secretion. This type (=“solid glandular type”) is taxonomically not well defined and includes taxa such as Trogulidae, Dicranolasmatidae, partly Nemastomatidae (Ortholasmatinae), and also certain Ischyropsalidoidea such as *Ischyropsalis* and some Ceratolasmatinae (Pabst 1953; Juberthie et al. 1991; Shear 2010; Schaider et al. 2010; Raspotnig, unpublished observations). Secretion of this type is not extractable or, at least, could so far not be extracted by different organic solvents (e.g., Schaider and Raspotnig 2009; Shear 2010). In all these taxa, emission of secretion cannot be recognized, and the natural mode of secretion release, if there is any, is unknown or highly speculative as outlined in “Introduction”. (2) A second glandular ecotype (“semi-volatile glandular type”) is characterized by the production of solid or viscous secretion that is liquefied in the course of the emission process and then may be applied in liquid form. This type of secretion includes semi-volatiles such as naphthoquinones and solids such as anthraquinones, which so far represented the only chemically known secretion components from the Dyspnoi. This type of secretion is characteristic for certain Nemastomatidae such as for genera *Paranemastoma* (Raspotnig et al. 2010), *Histricostoma*, and *Mediostoma* (Raspotnig, unpublished), and possibly others. (3) The volatiles produced by *Carinostoma* spp. characterize a third ecological type of scent glands among the Dyspnoi. We cannot assess the taxonomic distribution of this “volatile glandular type” for the time being, but preliminary investigations indicate that a number of nemastomatines, including all species of *Nemastoma* fall into this category (Raspotnig, unpublished). This third glandular type may be characterized as containing different volatiles in a semi-volatile naphthoquinone matrix. The absolute amounts of volatiles produced, as e.g., found for *C. ornatum*, are comparable to those from other Opiliones: Species of the laniatorean *Holoscotolemon* for instance (body length 3–4 mm) produce about 15 μg of secretion/individual (Raspotnig et al. 2011). This is about five to ten times more than the detected maximum amount in *C. ornatum* individuals (body length about 1.5 mm; body volume less than 1/10 of that in *Holoscotolemon*). Interestingly, and though not exactly quantified, *C. carinatum* appeared to produce the least amounts of secretion of all *Carinostoma* species: this was tentatively indicated by generally low peak areas or only trace amounts of acetophenone (compared to MHO peaks of *C. ornatum*) and underlined by the rather small size of scent glands as found in histological sections (Fig. 3).

### Volatile ketones from Dyspnoi: phylogenetic implications

Scent glands and their secretions have been the focus of opilionid chemosystematics for many years (Roach et al. 1980; Duffield et al. 1981; Hara et al. 2005; Caetano and Machado 2013), indeed representing a promising model system to study the evolution of secretion chemistry in exocrine glands in general (e.g., Raspotnig 2012). It certainly is an ambitious goal to logically and comprehensively explain the taxonomic distribution of chemical characters across the secretions of all extant opilionid taxa, but first approaches (mainly on Laniatores) are already available (e.g., Hara et al. 2005; Caetano and Machado 2013). For cyphophthalmid/palpatorean taxa, however, an enormous bulk of work still has to be processed to generate a comprehensive chemical database that allows a reliable reconstruction of the evolutionary history of secretion compounds. Dyspnoans, in particular, had virtually been excluded from the recently revived field of scent gland-based opilionid chemosystematics (e.g., Raspotnig 2012): the predominating dyspnoan trend toward increasing solidification of secretion (finally resulting in the “solid glandular type”) clearly impeded any chemosystematic approach, simply by a general inaccessibility of secretions (e.g., Shear 2010) or, at least, by methodological difficulties regarding their extraction (Raspotnig et al. 2010). Moreover, this trend toward solidification must be considered a highly derivative dyspnoan condition and is thus probably less valuable for reconstructing major trends of scent gland chemistry among the higher groups of Opiliones. From previous scent gland research, a few chemical classes of scent gland products have turned out to be well suited for a continuous and comprehensive reconstruction of opilionid chemistry: Such chemosystematically useful classes of compounds are certainly the laniatorean phenols and benzoquinones (Caetano and Machado 2013; Rocha et al. 2013a) as well as the cyphophthalmid/palpatorean naphthoquinones (see below). Another widespread and chemosystematically important class may be represented by scent gland-derived ketones (e.g., Ekpa et al. 1985; Raspotnig 2012). Indeed, all three *Carinostoma*-volatiles (6-methyl-5-hepten-2-one, octan-3-one, acetophenone) are ketones, and a comparably ketone-rich chemistry is meanwhile also indicated for *Nemastoma* and related genera (Raspotnig, unpublished). Thus, there is at least first evidence for further ketones among the Dyspnoi, possibly even representing the characteristic core chemistry of dyspnoan secretions of the volatile glandular type in general. To class the *Carinostoma* ketones (or the putative "volatile glandular-type" ketones) into the current chemosystematic picture of Opiliones, at least two major possible scenarios have to be considered: In a first scenario, the compounds may have evolved anew and represent products of a possibly independent biosynthetic route of the “volatile glandular type” in dyspnoan harvestmen. This scenario is simple and may be supported by the involvement of compounds such as 6-methyl-5-hepten-2-one—an exocrine compound relatively widespread in the secretions of other arthropods, e.g., in dolichoderine ants and certain coleopterans (Cavill et al. 1956; Schildknecht et al. 1976). Comparably, also octan-3-one has been reported from the secretions of various insects, in particular from the mandibular glands of myrmicine ants (e.g., Crewe et al 1969). In terms of a postulated “semiochemical parsimony” (sensu Blum 1996), such compounds have evolved several times independently in different taxonomic groups, obviously due to a common physico-chemical suitability as allomones.

Within a certain taxon, however, such compounds may show chemosystematic relevance, opening up the view onto a second scenario: scent gland-derived ketones seem to systematically “pervade” the secretions of Opiliones and are characteristic for all Cyphophthalmi so far investigated (methyl ketones), sclerosomatid Eupnoi (ethyl ketones), and a distinct group of gonyleptid Laniatores (vinyl ketones). These compounds have been detected in the Dyspnoi (methyl- and ethyl ketones) as well. While the laniatorean vinyl ketones clearly have an independent evolutionary origin, representing a synapomorphy of some gonyleptid subfamilies (Rocha et al. 2011, 2013b; Caetano and Machado 2013; Wouters et al. 2013), the idea of a common ancestry of the ketones from scent gland secretions of Cyphophthalmi/Palpatores is intriguing and has already been proposed (Raspotnig 2012). The results for *Carinostoma* (or as expected for the volatile glandular-type secretions in general) may support this view, though we are aware that the available data on cyphophthalmid/palpatorean chemistry base is still poor and biased (particularly for palpatorean secretions), currently not allowing too far-reaching conclusions. Though 6-methyl-5-hepten-2-one and octan-3-one are new compounds for the secretions of opilionids, they can—at least chemically—be assigned to methyl and ethyl ketones, as found in cyphopthalmids and sclerosomatids, respectively (Ekpa et al. 1985; Raspotnig et al. 2005, 2012; Jones et al. 2009). The third compound, acetophenone, also shows a ketone structure and even belongs to the pool of already reported cyphophthalmid secretion compounds (Raspotnig et al. 2005). Unfortunately, and contrasting with first insights into ketone biosynthesis in gonyleptids (Rocha et al. 2013b), nothing is known about the biosynthetic routes to ketone components in the Cyphophthalmi/Palpatores. A common route to these compounds in cyphophthalmids, Eupnoi and Dyspnoi, however, might indeed support an assumed common ketone ancestry. In this case, the *Carinostoma* volatiles represented a first chemosystematic link to the ketones of Cyphophthalmi and Sclerosomatidae and a first step toward a much more elaborated view on the chemosystematic overall picture of Opiliones.

### Evidence for common naphthoquinones

By contrast, the presence of naphthoquinones associated with the *Carinostoma* secretion was not too surprising: naphthoquinones are common in opilionid scent glands (except laniatorean glands) and may indeed represent a part of the phylogenetically old exocrine equipment of the Opiliones (Raspotnig 2012). In particular, 1,4-NQ and 6-MNQ have meanwhile been reported from cyphophthalmids of three different families hitherto analyzed (Raspotnig et al. 2005, 2012) and at least exemplarily from phalangiid Eupnoi (Wiemer et al. 1978) as well as from nemastomatid Dyspnoi (Raspotnig et al. 2010). The finding of 1,4-NQ and 6-MNQ in *Carinostoma* further underlines the status of these compounds as ancestral characters of cyphophthalmid/palpatorean scent gland secretions. The finding of 4-chloro-1,2-naphthoquinone in the secretions of *C. elegans* and (in traces) in *C. ornatum*, however, was unexpected since chloro-naphthoquinones have been considered a synapomorphy of cyphophthalmids and have not been detected in any other opilionid or in any other arthropod yet (e.g., Raspotnig et al. 2012). Nothing is known on their actual distribution among dyspnoans, but the occurrence of these exceptional compounds in *Carinostoma* implies a common ancestry with Cyphophthalmi as well.

Functionally, the involvement of naphthoquinones in an effective operation of the *Carinostoma* secretion is still not clear. Their origin in the scent glands of *Carinostoma* is virtually certain: In *P. quadripunctatum*, a large nemastomatid close to *Carinostoma* (Schönhofer et al. 2013) these naphthoquinones are major components of the secretions, and scent gland reservoirs even appear reddish by naphthoquinones after excision. We assume an analogous situation to be true for species of *Carinostoma*. The process of secretion emission, however, may clearly differ between these two genera: When *Paranemastoma* applies its secretion in the course of natural operation, naphthoquinones (and anthraquinones) are liquefied by enteric fluid via a rather intricate mechanism, probably involving the conduction of enteric fluid into scent gland reservoirs and the subsequent discharge of a quinone-loaded mixture (Schaider et al. 2011). In *Carinostoma*, however, a particular liquefaction process of the naphthoquinones by mixing up with enteric fluid has never been observed and may not be necessary either. It is very likely that the naphthoquinones are already dissolved in the volatile ketone fraction in the reservoirs and that an already accomplished mixture is ejected.
